# Lethal Cord Entanglement

**DOI:** 10.7759/cureus.31047

**Published:** 2022-11-03

**Authors:** Punit Hans, Gunjan Gunjan

**Affiliations:** 1 Obstetrics and Gynaecology, Patna Medical College, Patna, IND

**Keywords:** antenatal care, twin pregnancy, umbilical cord entanglement, monochorionic monoamniotic twins, monoamniotic twins

## Abstract

Cord entanglement and conjoined twins are unique complications of monoamniotic monochorionic pregnancy. This case report describes a case of monoamniotic twins' intrauterine demise due to lethal cord entanglement. A 26-year-old unbooked primigravida was reported in the emergency labor room at 32 weeks with the complaint of loss of fetal movements since the previous day. On obstetric examination, the uterus appeared enlarged and discordant with the gestational age, multiple fetal parts were felt on palpation, and the pelvic grip was empty. Doppler could not detect any fetal heartbeat. The emergency obstetric sonography scan showed twins, the first in breech and the second in vertex presentation. Fetal heart sound was not found for either of the twins. The patient was counseled, and the decision was taken to terminate the pregnancy by lower-segment cesarean section. During the cesarean to deliver the babies, monochorionic and monoamniotic pregnancy was confirmed.

## Introduction

Monochorionic monoamniotic gestation is known as the least common type of twin pregnancy. In a monoamniotic twin pregnancy, the twins share a single placenta, amnion, and chorion. The zygote division after fertilization decides the chorionicity and amniotic status of the pregnancy. Division on days 8-12 leads to monochorionic monoamniotic placentation. Factors affecting the time of division are not yet apparent. In vitro fertilization seems to play a role as it increases the frequency of monozygotic twinning [1-3]. Monoamniotic twin gestations are found in approximately 5% of monochorionic and 1% of twin pregnancies. Around one in 10,000 spontaneously conceived pregnancies involves monoamniotic twins [4-6]. Cord entanglement and conjoined twins are some complications unique to monoamniotic monochorionic pregnancy. This case report describes a case of monoamniotic twins' intrauterine demise due to lethal cord entanglement.

## Case presentation

A 26-year-old unbooked primigravida reported in the emergency labor room at 32 weeks with the complaint of loss of fetal movements since the previous day. She was sure of her last menstrual period. She had acknowledged her pregnancy when a urine pregnancy test showed a positive result two days after she missed her period, and this was a spontaneous conception after one year of marriage. She had three antenatal visits at the local health care center before reporting to our emergency labor room. She had complained of excessive nausea and vomiting during the first trimester; otherwise, her antenatal period had been uneventful. She had two ultrasound scan reports for fetal well-being; the earliest scan was at 12 weeks and the latest was at 28 weeks. Twin pregnancy with adequate amniotic liquor and a fundal-anterior placenta was mentioned in the reports. There were no comments on chorionicity.

On general examination, she had mild pallor, bipedal edema, a pulse rate of 98 beats per minute, and a blood pressure of 150/90 mmHg. On obstetric examination, the uterus appeared enlarged and discordant with the gestational age, multiple fetal parts were felt on palpation, and the pelvic grip was empty. Doppler could not detect any fetal heartbeat. A vaginal examination revealed that the os was closed, and the cervix was posterior.

The emergency obstetric sonography scan showed twins, the first in the breech and the second in vertex presentation. Fetal heart sound was not found in either of the twins. The patient was counseled, and the decision was taken to terminate the pregnancy by lower-segment cesarean section.

**Figure 1:**
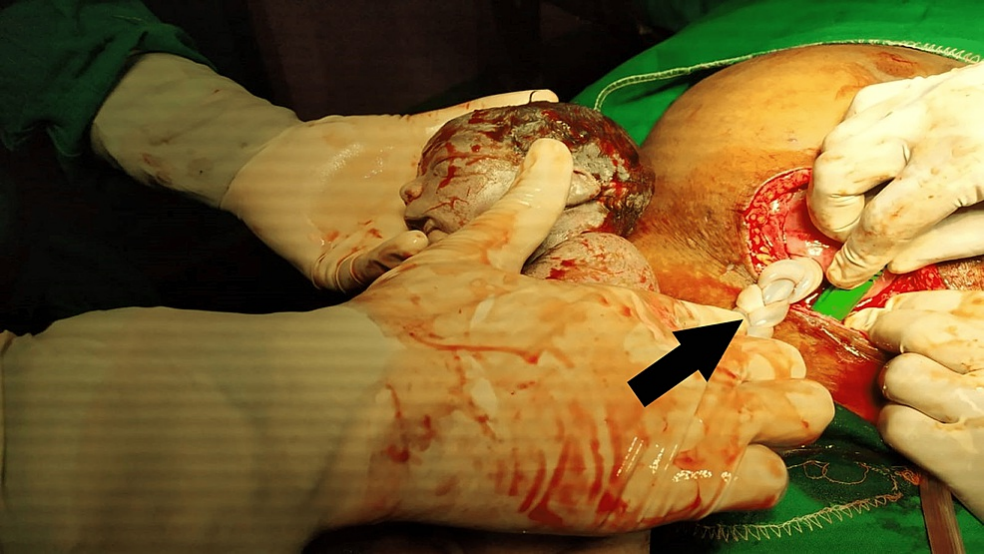
Intrauterine demise with thrombosed entangled cord Black arrow shows thrombosed cord after entanglement

**Figure 2:**
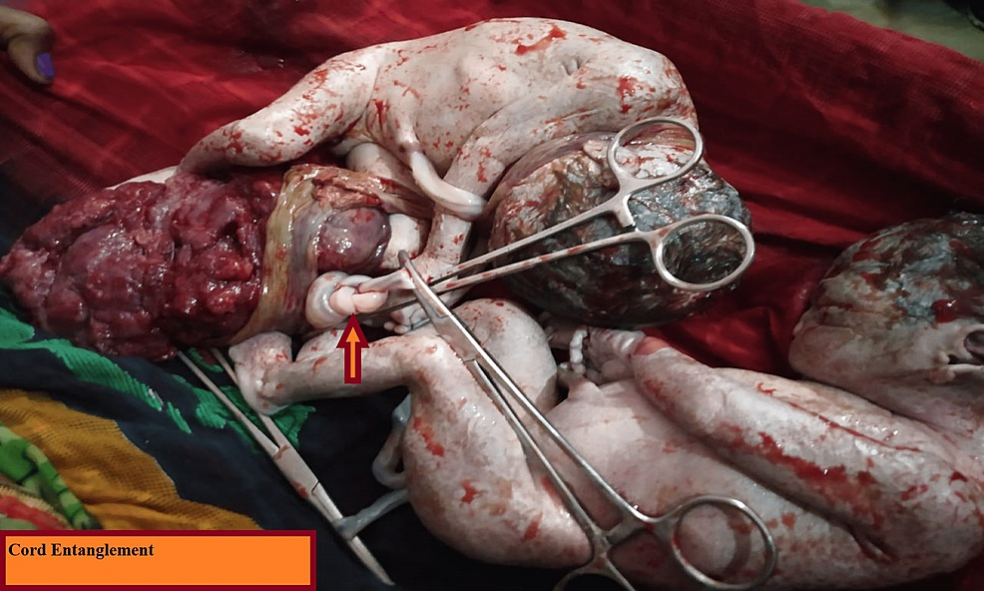
Dead twins with cord entanglement Arrow shows cord entanglement

During the cesarean to deliver the babies, monochorionic and monoamniotic pregnancy was confirmed by the following features: a single placenta, where the two cords were inserted centrally within 6 cm from each other, the absence of an intertwining membrane, and same-sex babies. The entanglement of the cords, which we suspected was the cause of intrauterine death, was unique. Figures 1, 2 show intrauterine twin demise with entangled cords.

Post-cesarean, the patient was moved to the recovery ward. Milk suppression treatment was given, the patient was counseled, and her post-partum period was uneventful.

## Discussion

Proper antenatal care and the timely detection of abnormality with timely intervention, which were altogether absent in this case, are critical in managing monochorionic monoamniotic pregnancies.

Monoamniotic monochorionic pregnancies can be diagnosed prenatally in the first trimester. The features include a single placenta that is not fused and no intertwining membrane between same-sex twins [7]. Fetal mortality is very high in monoamniotic gestations, around 12-23% [8-10], due to the complications unique to this gestation, such as cord entanglement and conjoined twins, and other complications associated with different types of twin pregnancies.

Cord entanglement, when found on ultrasonography, is pathognomonic for monoamniotic twins. It can be seen as early as 12-13 weeks of pregnancy and diagnosed after visualization of intertwined umbilical cords, which, upon Doppler insonation, show different fetal heart rates [5,11-13]. In the first trimester, cord entanglements may appear loose but can tighten at any time [14]. According to some studies, intermittent occlusion of umbilical blood vessels may be associated with neurological morbidity, while severe prolonged occlusion can be fatal [14,15]. Inpatient antepartum fetal monitoring and early delivery have been found to reduce the perinatal death rates in monoamniotic twin pregnancies. Monoamniotic twin pregnancies should be delivered by cesarean section between 32 and 34 weeks [16,17].

## Conclusions

Prenatal care is of utmost importance for monochorionic monoamniotic pregnancies. Antepartum fetal surveillance should be started early, at 24-28 weeks, and inpatient monitoring seems more beneficial. Delivery should be by cesarean section, and tertiary health care with the facility of a special newborn care unit should be available as the risk of prematurity and congenital anomalies is extremely high.
